# Relation between aphasia and arcuate fasciculus in chronic stroke patients

**DOI:** 10.1186/1471-2377-14-46

**Published:** 2014-03-08

**Authors:** Hyung Jun Tak, Sung Ho Jang

**Affiliations:** 1 Department of Physical Medicine and Rehabilitation, College of Medicine, Yeungnam University, 317-1, Daemyungdong, Namku, Taegu 705-717, Republic of Korea

**Keywords:** Aphasia, Stroke, Arcuate fasciculus, Diffusion tensor tractography

## Abstract

**Background:**

The role of the arcuate fasciculus (AF) in the dominant hemisphere in stroke patients with aphasia has not been clearly elucidated. We investigated the relation between language function and diffusion tensor tractography (DTT) findings for the left AF in chronic stroke patients with aphasia.

**Method:**

Twenty five consecutive right-handed stroke patients with aphasia following lesions in the left hemisphere were recruited for this study. The aphasia quotient (AQ) of Korean-Western Aphasia Battery was used for assessment of language function. We measured values of fractional anisotropy (FA), apparent diffusion coefficient (ADC), voxel number of the left AF. We classified patients into three groups: type A - the left AF was not reconstructed, type B - the left AF was discontinued between Wernicke’s and Broca’s areas, and type C – the left AF was preserved around the stroke lesion.

**Results:**

Moderate positive correlation was observed between AQ and voxel number of the left AF (*r* = 0.471, *p* < 0.05). However, no correlation was observed between AQ and FA (*r* = 0.275, *p* > 0.05) and ADC values (*r* = -0.286, *p* > 0.05). Significant differences in AQ scores were observed between the three types (*p* < 0.05); the AQ score of type C was higher than those of type A and B, and that of type B was also higher than that of type A (*p* < 0.05).

**Conclusion:**

According to our findings, the remaining volume of the left AF, irrespective of directionality and diffusivity, showed moderate positive correlation with language function in chronic stroke patients with aphasia. Discontinuation or non-construction of the left AF was also an important factor for language function.

## Background

Aphasia is one of the most common sequelae of stroke. Approximately 24%-38% of stroke patients have been reported to suffer from aphasia during acute stage [1-4]. Although patients with aphasia can show some degree of spontaneous recovery, this improvement of aphasia is observed mainly during the first three months after stroke onset [2,3,5-7]. Consequently, approximately 10-18% of stroke patients are known to suffer from aphasia during chronic stage [1,6-8].

Since identification of the arcuate fasciculus (AF) as the neural tract connecting Broca’s and Wernicke’s areas by Von Monakow, the AF has been regarded as an important neural tract for language [9]. Previous studies of the AF using intra-operative mapping techniques, brain CT, conventional brain MRI, or functional neuroimaging techniques have been conducted [10-12]. However, because it cannot be clearly discriminated from adjacent structures, many difficulties have been encountered in the effort to obtain an accurate estimation of the AF.

By contrast, diffusion tensor tractography (DTT), derived from diffusion tensor imaging (DTI), allows for identification and evaluation of the AF in 3-D images [13,14]. In recent years, many studies using DTT for evaluation of the AF have been conducted for research on aphasia in stroke patients [15-23]. Several of these studies have demonstrated that impairment of the AF in the dominant hemisphere could play an important role in aphasia [15,16,18,19,23]. However, the role of the AF in the dominant hemisphere in stroke patients with aphasia has not been clearly elucidated.

In the current study, we attempted to investigate the relation between language function and DTT findings of the left AF in chronic stroke patients with aphasia.

## Methods

### Subjects

Twenty five consecutive right-handed patients (mean age 46.0 ± 11.6 years) were recruited according to following inclusion criteria: (1) first-ever stroke, (2) age range: 20 ~ 69 years, (3) stroke lesion was located in the left hemisphere, (4) aphasia: aphasia quotient (AQ) scores on Korean-Western Aphasia Battery (K-WAB) below 92.8, (5) DTI scanning and K-WAB were performed after three months from stroke onset, (6) no history of stroke, head trauma, or psychiatric disorder, and (7) no previous stroke lesion on the brain MRI which was taken at stroke onset [24]. This study was performed retrospectively and the study protocol was approved by the Institutional Review Board of the Yeungnam university hospital.

### Evaluation of language function

The AQ of K-WAB was conducted for assessment of language dysfunction at a chronic stage of stroke (mean 229.2 ± 159.4 days after onset). Reliability and validity of K-WAB have been well-established [24,25].

### Diffusion tensor imaging

DTI data were acquired at an average of 181.5 ± 75.3 days after onset using the 1.5-T Philips Gyroscan Intera system (Hoffman-LaRoche, Mijdrecht, Netherlands), equipped with a synergy-L Sensitivity Encoding (SENSE) head coil and utilizing a single-shot, spin-echo planar imaging pulse sequence. Sixty-seven contiguous slices were acquired for each of the 32 non-collinear diffusion-sensitizing gradients. Imaging parameters were as follows: acquisition matrix = 128 128 matrix, field of view = 221 221 mm^2^, TE = 76 ms, TR = 10,726 ms, SENSE factor = 2; EPI factor = 67 and b = 600 mm^2^s^-1^; NEX = 1; and a slice thickness of 2.3 mm.

**Figure 1:**
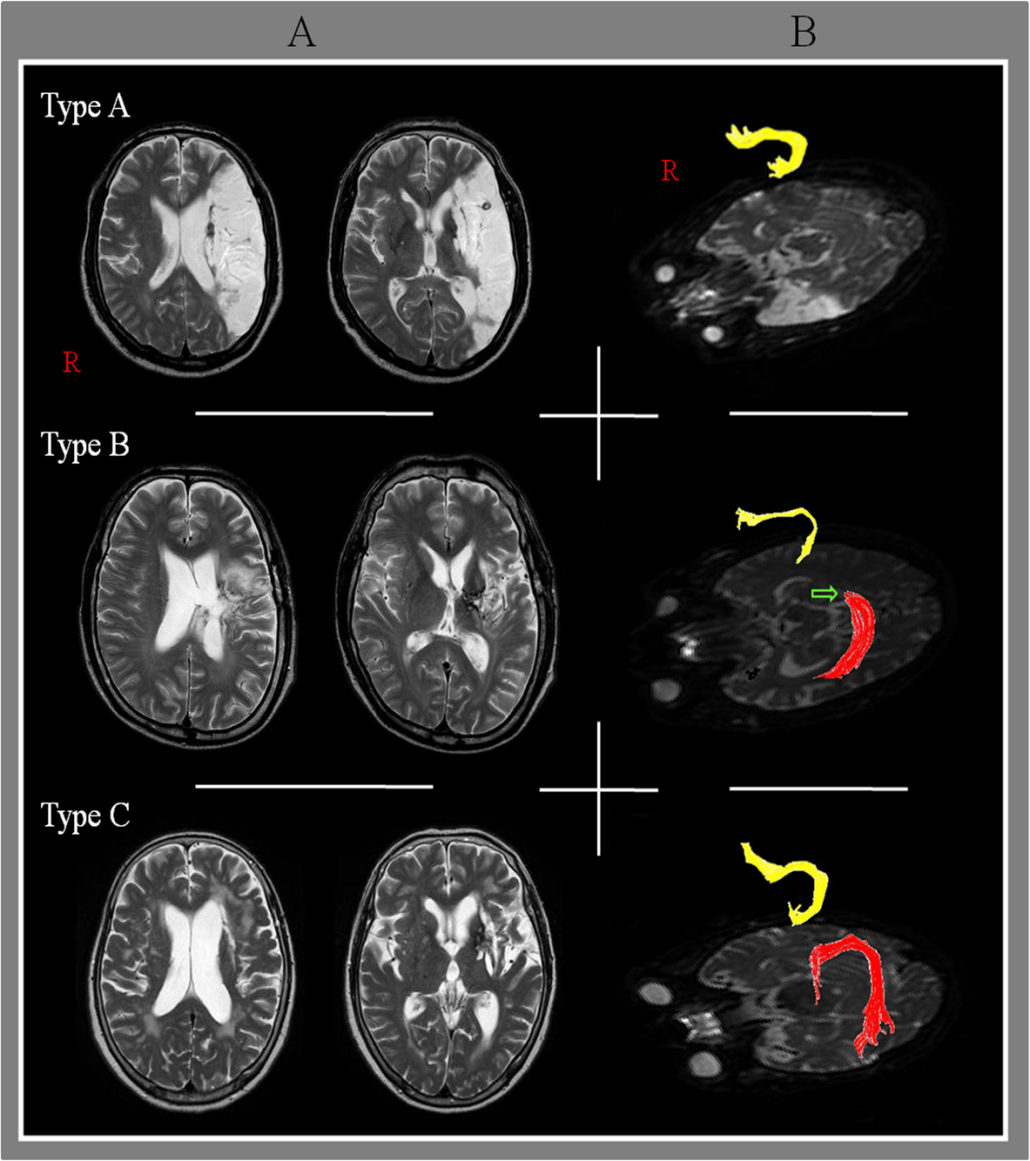
**Classification of aphasia patients according to severity of the arcuate fasciculus impairment. (A)** T2-weighted brain MRI in chronic stroke patients with aphasia, **(B)** diffusion tensor imaging tractogrophy for the arcuate fasciculus (AF) (Right AF: yellow color, left AF: red color, arrow: discontinuation).

Removal of eddy current-induced image distortions using affine multi-scale two-dimensional registration was performed at the Oxford Centre for Functional Magnetic Resonance Imaging of Brain (FMRIB) Software Library (FSL; http://www.fmrib.ox.ac.uk/fsl) [26]. DTI-Studio software (CMRM, Johns Hopkins Medical Institute, Baltimore, MD, USA) was used for evaluation of the left AF, which was based on the fiber assignment continuous tracking (FACT) algorithm and the multiple regions of interest (ROIs) approach. By using Nucifora’s method, we placed two ROIs for tracking of the AF, i.e. the seed ROI in the posterior parietal area of the superior longitudinal fascicle and the target ROI in the posterior temporal lobe [27-29]. Termination criteria used for fiber tracking was FA < 0.2 and angle < 60 [23].We measured the values of fractional anisotropy (FA), apparent diffusion coefficient (ADC), and voxel number of the left AF. We then classified patients into three groups, according to the severity of the AF impairment: type A - the left AF was not reconstructed, due to severe injury and degeneration (Figure 1). We confirmed non-reconstruction of the left AF by lowering the FA value to 0.1 and by placement of only one ROI along the left AF pathway. In addition, we confirmed severe degeneration of the left AF on the color map. Type B - the left AF was discontinued between Wernicke’s and Broca’s areas, type C – the left AF was preserved around the stroke lesion, i.e., the tract originating from Wernicke’s area and passing around the lesion to Broca’s area.

### Measurement of volume of stroke lesion

Volume of stroke lesion was measured on T2-weighted MRI images using a picture-archived communication system (PACS, Marotech, Korea). We measured maximum width (X), length (Y), and height (Z) of the lesion at the level where stroke lesion could be clearly observed. Lesion volume was calculated according to the formula [30]:

$$\begin{array}{l} \mathrm{Lesion}\;\mathrm{volume}\;\left(\mathrm{mV}\right)=\frac{4}{3}\;\times \;\frac{1}{16}\;\times \pi \times X\left(\mathrm{cm}\right)\\ \;\;\;\;\;\times Y\left(\mathrm{cm}\right)\times Z\left(\mathrm{cm}\right). \end{array}$$

### Statistical analysis

Statistical Package for the Social Sciences for windows (SPSS version 12.0 K, SPSS Korea) was used in performance of all statistical analyses. The Mann-Whitney test was performed for comparison of significant differences of AQ, age, volume of stroke lesion (mV), DTI evaluation time post onset (days), and K-WAB evaluation time post onset (days) between the three types of the left AF. The Mann-Whitney test was also used for comparison of differences in DTT parameters (FA, ADC, and voxel number) between type B and type C. Pearson’s correlation analysis was employed for estimation of significant correlation of AQ with DTT parameters (FA, ADC, and voxel number). The level of statistical significance was set at *p* <0.05.

## Results

Demographic and DTT findings are summarized in Table 1. Twenty-five patients were classified into three groups according to the DTT type for the AF. Six patients belonged to the type A group (four males; mean age 49.3 ± 7.8 years; range 41 to 63 years), eleven patients to the type B group (six males; mean age 49.6 ± 9.0 years; range 37 to 66 years), and eight patients (four males; mean age 38.6 ± 14.5 years; range 20 to 63 years) to the type C group. No difference in distribution of age, DTI scanning time from onset, and K-WAB evaluation time from onset was observed between the three AF types (*p* > 0.05). However, lesion volume of the type A group was greater than those of type B and C groups (*p* < 0.05) although there was no difference between the type B and C groups (*p* > 0.05). Significant differences in AQ scores were observed between the three types (*p* < 0.05); AQ score of the type C group was higher than those of type A and type B, and that of type B was also higher than that of type A (*p* < 0.05). With regard to FA, ADC, and voxel number of the left AF, no significant differences were observed between the type B and C groups (*p* > 0.05).

**Table 1:** Demographic and diffusion tensor tractography data for the left arcuate fasciculus

**Variables**	**Total**	**Diffuse tensor tractography type**		
		**A**	**B**	**C**
Patient number (male: female)	25 (14:11)	6 (4:2)	11 (6:5)	8 (4:4)
Age	46.0 (11.6)	49.3 (7.8)	49.6 (9.0)	38.6 (14.5)
Days to diffusion tensor imaging	181.5 (75.3)	156.3 (75.0)	187.9 (65.4)	191.5 (92.7)
Days to Korean-western aphasia battery	229.2 (159.4)	181.7 (103.7)	192.5 (67.8)	315.3 (245.7)
Stroke lesion volume (mV)	24.8 (23.7)	58.8 (25.4)	15.0 (7.8)	12.8 (8.8)
Aphasia quotient	43.5 (29.4)	11.1 (6.0)	41.6 (24.5)	70.4 (18.9)
Fractional anisotropy	0.44 (0.06)	-	0.42 (0.30)	0.46 (0.78)
Apparent diffusion coefficient	0.82 (0.41)	-	0.82 (0.03)	0.81 (0.05)
Voxel number	7.85 (3.32)	-	6.37 (2.34)	9.89 (3.52)

Values indicate mean (± standard deviation).

Moderate positive correlation was observed between AQ and voxel number of the left AF (*r* = 0.471, *p* < 0.05) [31]. However, no correlation was observed between AQ and FA (*r* = 0.275, *p* > 0.05), and ADC values (*r* = -0.286, *p* > 0.05) (Table 2).

**Table 2:** Correlation between aphasia quotient with diffusion tensor tractography parameters of the left arcuate fasciculus

	**Aphasia quotient**	
	**Correlation (*r*)**	***p*-value**
Fractional anisotropy	0.275	0.255
Apparent diffusion coefficient	−0.286	0.236
Voxel number	0.471	0.042*

*p < 0.05.

## Discussion

In this study, using DTT, we analyzed the status of the left AF in chronic stroke patients with aphasia and investigated relations between AQ and DTT findings of the left AF. Our results can be summarized as follows: 1) AQ showed moderate positive correlation with voxel number of the left AF. However, it did not show correlation with FA and ADC values of the left AF. 2) AQ differed according to the discontinuation or non-construction of the left AF; specifically, poorer AQ scores were observed for patients who showed discontinuation of integrity or non-construction of the left AF on DTT, compared with patients who did not show those findings. FA value represents the degree of directionality of microstructures (e.g., axons, myelin, and microtubules), and ADC value indicates the magnitude of water diffusion [14,32]. The opposite correlation of FA (*r* = 0.275) and ADC (*r* = -0.286) with AQ appear to be attributed to these characteristics of FA and ADC values [14,32]. In contrast, the voxel number indicates the volume of the remaining AF [33,34]. Therefore, our results appear to indicate that the voxel number of the left AF reflected language function of patients, irrespective of directionality and diffusivity of the AF. On the other hand, discontinuation or non-construction of the left AF was an important factor for language function in these patients. Non-reconstruction of the left AF in the type A group appears to indicate severe degeneration of the left AF following severe injury of the left AF at stroke onset.

Following introduction of DTI, several studies reported on the relationship of AF finding on DTI and language function in stroke patients with aphasia [15,16,18,19,23]. In 2008, Breier et al reported FA values on DTT of the left AF and superior longitudinal fasciculus scanned at chronic stage (1-72 months), showed correlation with repeatability of language in 20 stroke patients with aphasia [15]. In 2009, Hosomi et al reported on comparison of the asymmetry of FA value and fiber number between the right and left AF on DTT taken within two days from onset in 13 patients with left middle cerebral artery infarcts. Significant loss of fiber number of the left AF, compared with the right AF, was observed in the group of patients with aphasia, irrespective of change of FA value at discharge (13-52 days after onset) [16]. In 2010, Zhang et al reported on a decrease in FA value and fiber number of the left AF in 10 patients with conduction aphasia [18]. Using DTT, they also observed injuries of the left AF in these patients. Subsequently, Marchina et al [2010], who estimated the volume of three language-related neural tracts (the AF, extreme capsule, and uncinate fasciculus, which were affected by a stroke lesion), found that lesion loads of the AF were predictive of language function in terms of rate, informativeness, efficacy of speech, and naming ability in 23 chronic stroke patients with aphasia [19]. A recent study by Kim et al [2012] reported that the integrity of the left AF was an important factor in prediction of prognosis of language function in stroke patients with aphasia [23]. Therefore, our results coincided with findings from the previous studies described above, showing correlation of language function of stroke patients with aphasia with the volume of the remaining left AF and preservation of integrity of the left AF was found to be important for language function in stroke patients [16,19,23]. However, our result showing that FA value of the left AF did not show correlation with language function was not completely consistent with results of previous studies reporting controversial results [15,18]. We believe that this discordance might be attributed to the characteristics of FA value, which represents the degree of directionality of the AF [14,32].

## Conclusions

In conclusion, according to our findings, the remaining volume of the left AF, irrespective of directionality and diffusivity, showed moderate positive correlation with language function in chronic stroke patients with aphasia. Discontinuation or non-construction of the left AF was also an important factor for language function. One of the limitations of this study was the small number of subjects. Because this study was conducted retrospectively, we could not conduct a detailed evaluation of the language function of patients. In addition, we did not control the education level of patients and the volume of stroke lesion volume which can affect the language function. The fact that we did not analysis the other neural tracts which are involved in language function is an another limitation of this study [35]. Therefore, further prospective studies to overcome these limitations should be encouraged. On the other hand, limitations of DTT should be considered: the fiber tracking technique is operator-dependent, and regions of fiber complexity and crossing might prevent full reflection of the underlying fiber architecture [36,37].

## Abbreviations

AF: Arcuate fasciculus; DTT: Diffusion tensor tractography; DTI: Diffusion tensor imaging; K-WAB: Korean-Western Aphasia Battery; FMRIB: Functional Magnetic Resonance Imaging of Brain; FA: Fractional anisotropy; ADC: Apparent diffusion coefficient.

## Competing interests

The authors declare that they have no competing interests.

## Authors’ contributions

HJT participated in the design of the study, collection and analysis of data, and drafting the manuscript. SHJ participated in the design of the study, funding, and writing the manuscript. Both authors read and approved the final manuscript.

## Pre-publication history

The pre-publication history for this paper can be accessed here:

http://www.biomedcentral.com/1471-2377/14/46/prepub
